# Septicemia, necrotizing fasciitis, and peritonitis due to *Vibrio vulnificus* treated with early use of polymyxin B hemoperfusion in a patient undergoing CAPD: a case report

**DOI:** 10.1186/s12882-020-01772-2

**Published:** 2020-04-09

**Authors:** Jae Young Kim, Young Su Joo, Sangmi Lee, Ji Young Lee, Jung Tak Park, Seong Hyeok Han, Tae-Hyun Yoo, Shin-Wook Kang

**Affiliations:** 1 Department of Internal Medicine, College of Medicine, Institute of Kidney Disease Research, Yonsei University College of Medicine, Seoul, Republic of Korea; 2 Department of Internal Medicine, College of Medicine, Severance Biomedical Science Institute, Brain Korea 21 PLUS, Yonsei University, Seoul, Republic of Korea

**Keywords:** *Vibrio vulnificus*, Septicemia, Peritonitis, Peritoneal dialysis, Hemoperfusion, Polymyxin B immobilized fiber

## Abstract

**Background:**

*Vibrio vulnificus* infection is a rare but fatal foodborne illness. Here, we report a case of *Vibrio vulnificus* peritonitis followed by severe septicemia in a patient undergoing continuous ambulatory peritoneal dialysis (CAPD) who was treated with hemoperfusion using polymyxin B immobilized fiber.

**Case presentation:**

A 63-year-old man undergoing CAPD was admitted to the emergency room due to general weakness, fever, and abdominal pain with hazy dialysate. Two days before admission, he had eaten raw fish. Initial laboratory tests including peritoneal fluid analysis suggested peritonitis. Despite empirical intraperitoneal antibiotic treatment, his fever did not subside, and multiple vesicles on the extremities newly appeared. The result of initial peritoneal fluid culture and blood cultures reported *Vibrio vulnificus* as the most likely causative pathogen. Hemoperfusion with polymyxin B immobilized fiber was performed to control gram-negative bacterial septicemia with antibiotics targeting the pathogenic organism. The patient recovered completely and was discharged without complications.

**Discussion and conclusion:**

Suspicion of *Vibrio vulnificus* infection in susceptible immunocompromised patients is important for early diagnosis and prompt management. Peritonitis should be noted as a clinical manifestation of *Vibrio vulnificus* infection in CAPD patients, and polymyxin B hemoperfusion along with proper antibiotics could be considered as a treatment option.

## Background

*Vibrio vulnificus* is a gram-negative rod-shaped bacterium found in warm marine environments. The main clinical manifestations of *Vibrio vulnificus* infection are gastrointestinal illness, wound infection, and primary septicemia. In particular, oral ingestion of or wound exposure to raw sea products in immunocompromised individuals, such as patients with liver cirrhosis, iron overload disorders, diabetes mellitus, steroid use, and chronic kidney disease (CKD), can lead to sepsis and fatal outcomes [1, 2]. Even with early diagnosis and prompt treatment, the mortality rate is extremely high. Here, we report a case of peritonitis followed by severe septicemia caused by *Vibrio vulnificus* in a patient undergoing continuous ambulatory peritoneal dialysis (CAPD) who was successfully treated by antibiotics and hemoperfusion using polymyxin B immobilized fiber.

## Case presentation

**Fig. 1:**
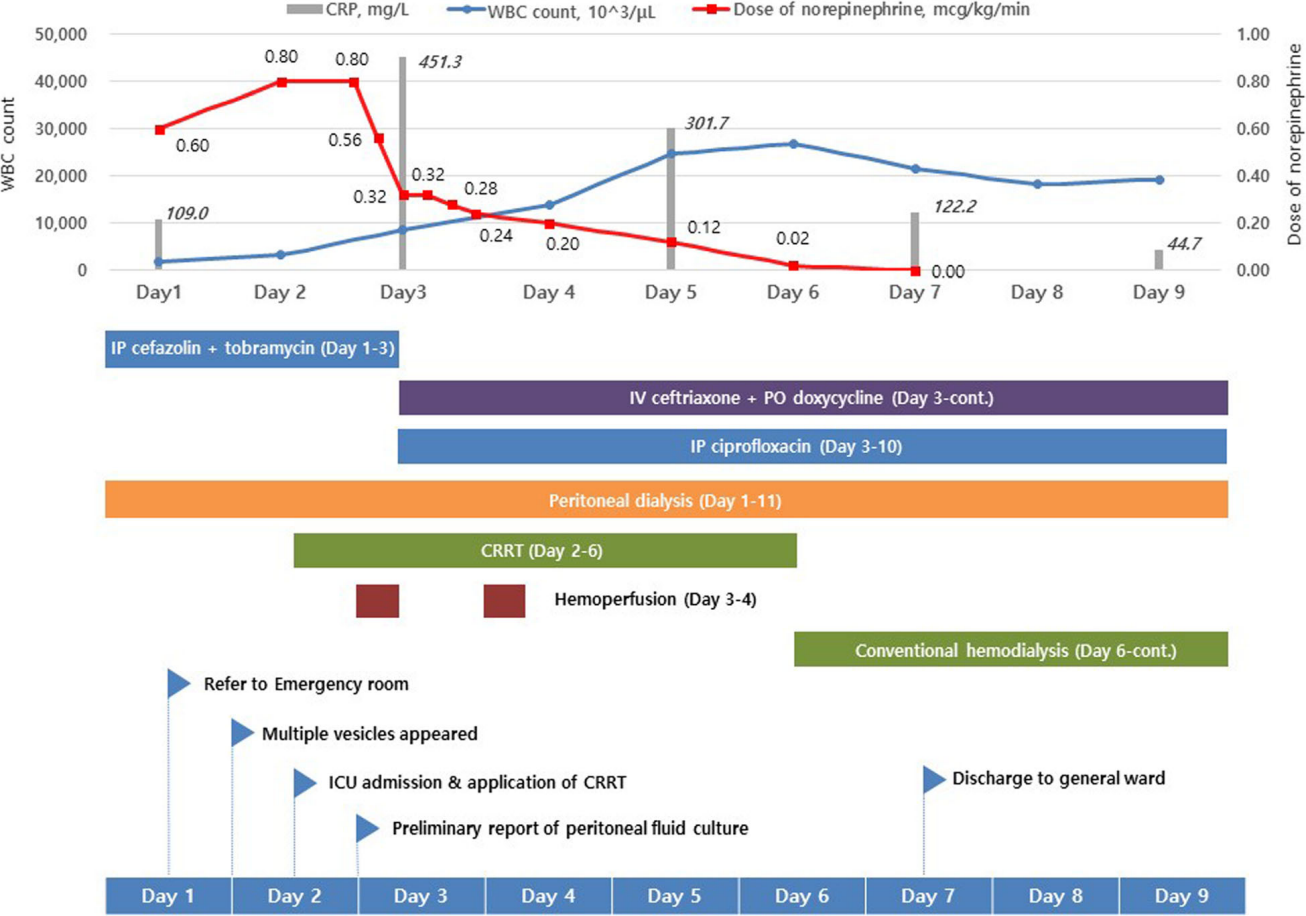
The time sequence of key events. Abbreviations: WBC, white blood cell; CRP, C-reactive protein; IP, intraperitoneal; IV, intravenous; PO, per os; CRRT, continuous renal replacement therapy; ICU, intensive care unit

A 63-year-old man who had undergone CAPD for 11 years was admitted to the emergency room due to general weakness, fever, and abdominal pain with hazy dialysate. He was an East Asian male, an office worker, who was 1 meter and 70 centimeters tall weighing 67.0 kg. His CAPD regimen consisted of a, 4 times a day, conventional 1.5% glucose anhydrous based dialysis solution containing lactate. The patient had been anuric for several years. Erythropoiesis-stimulating agents nor intravenous iron replacement had not been prescribed during the last month. However, he had been constantly receiving oral iron replacement. Two days before admission, he ate a sliced raw skate (thornback ray), and abdominal pain and diarrhea developed on the next day. The underlying cause of his end-stage renal disease (ESRD) was chronic glomerulonephritis, and there was no history of liver disease, diabetes mellitus, or steroid use. On admission, his body temperature was 39.5 °C, blood pressure 116/62 mmHg, and pulse rate 99 beats/min. There was direct and rebound tenderness on the whole abdomen. No definite skin lesions were discovered on any part of his body at initial evaluation, and the exit site of the peritoneal dialysis catheter was clean. Serum laboratory findings were as follows: white blood cell (WBC) count 1930/μL, hemoglobin level 8.0 g/dL, aspartate/alanine transaminase levels 37/72 IU/L, serum iron level 36.0 μg/dL, transferrin saturation 22.0%, ferritin level 745.6 μg/L, C-reactive protein (CRP) level 109 mg/L, and procalcitonin level 65.89 ng/mL. Peritoneal fluid analysis revealed that WBC count was 3400/μL with 80.1% of PMN cells. The laboratory results are summarized in Table 1. Peripheral blood and peritoneal fluid cultures were performed, and the patient was empirically treated with initial loading doses of intraperitoneal cefazolin 1000 mg and tobramycin 60 mg followed by maintenance doses of cefazolin 250 mg per each PD (peritoneal dialysis) and tobramycin 30 mg once a day. On the hospital day 2, his body temperature remained elevated at 38.3 °C, and his blood pressure decreased to 78/60 mmHg. The follow-up laboratory test results showed that plasma sodium level was 130 mmol/L, potassium level 6.2 mmol/L, chloride level 91 mmol/L, bicarbonate level 14.4 mmol/L, and arterial blood pH 7.351. In addition, multiple vesicles with annular erythema and peripheral edema appeared on both lower extremities. Even after appropriate fluid supplementation, the patient remained in persistent shock, and the inotropic agent dose was increased to maintain the patient’s blood pressure. The patient was consequently transferred to the intensive care unit (ICU) for close monitoring, and continuous renal replacement therapy (CRRT) was initiated. At the time of ICU admission, the patient’s SOFA (Sequential Organ Failure Assessment) score was 12, APACHE-II (Acute Physiology and Chronic Health Evaluation-II) score was 25, and SAPS-II (Simplified Acute Physiology Score-II) score was 53. Continuous venovenous hemodiafiltration was prescribed for CRRT. Treatment was delivered by a Prisma Flex machine using a ST100 (surface area, 1.0 m^2^) filter set, which contain a polyacrylonitrile AN 69 membrane (Gambro). Blood flow rate was started at 100 mL/minute and gradually increased to 150 mL/minute. Effluent volume was set to achieve a clearance of 40 mL/kg/hour. Replacement and dialysate volumes were set using the 1:1 balanced-predilution method. On the hospital day 3, the preliminary result of the initial peritoneal fluid culture reported *Vibrio vulnificus* as the most likely causative pathogen. Considering the preliminary culture study report, systemic intravenous ceftriaxone (2000 mg twice a day) and oral doxycycline (100 mg twice a day) were initiated, and the intraperitoneal antibiotics were immediately changed to ciprofloxacin (200 mg per each PD). With the change of antibiotics, hemoperfusion with polymyxin B immobilized fiber (Toraymyxin PMX-20R, Toray Medical, Tokyo, Japan) was concomitantly initiated, since gram-negative bacterial infection seemed to be the cause of the patient’s hemodynamic instability. In addition to CRRT, 2 hours of hemoperfusion was applied daily for two consecutive days. Approximately 3 h after hemoperfusion commencement, the patient’s electrolyte imbalance and metabolic acidosis were ameliorated, and his blood pressure stabilized, allowing a decrease in the dose of the inotropic agents without compromising his hemodynamic stability. Although the skin lesions on both lower extremities showed slight signs of aggravation, the patient did not exhibit any signs of compartment syndrome, such as motor weakness, altered sensation, or pulselessness. Based on these findings, the orthopedic surgeons decided that it was not necessary to have the patient undergo emergent fasciotomy. Therefore, only sterile dressings on the wet wounds were carried out daily during the ICU stay. The final reports of the bacterial cultures performed at the time of admission confirmed the growth of *Vibrio vulnificus* from both the peritoneal fluid and the peripheral blood with sensitivity to cefotaxime, ceftazidime, ciprofloxacin, and tetracycline. On hospital day 5, the WBC count in the peritoneal fluid decreased to 57/μL with 3% of PMN cells, and the CRP level decreased from 451.3 to 301.7 mg/L. CRRT was discontinued on the hospital day 6, and the patient was sent to the general ward on the day after CRRT discontinuation. Treatment with antibiotics, including intravenous ceftriaxone and oral doxycycline, was continued for two more weeks. Peritoneal dialysis was continued until hospital day 11 on which the PD catheter was removed. The patient recovered without significant complications and was discharged on the 46th day of hospitalization. The time sequence of key events is presented in Fig. 1.

**Table 1:** Laboratory examination results and inotropic dosage

	Day 1	Day 2 11 h prior HP	Day 3 3 h after HP	Day 4	Day 5	Day 6	Day 7	Day 8	Day 9
**Laboratory data**									
WBC count, 10^2/μL	19.3	32.4	85.5	138.3	246.6	269.1	216.5	181.6	192.9
Hemoglobin, g/dL	8.0	10.4	8.6	7.3	8.3	8.7	9.0	8.2	8.1
Platelet count, 10^3/μL	103.0	54.0	57.0	53.0	57.0	67.0	73.0	72.0	95.0
BUN, mg/dL	62.6	62.2	31.1	20.7	20.2	34.5	29.9	50.0	72.3
Cr, mg/dL	12.9	10.6	4.0	2.0	1.4	1.4	1.4	2.5	3.8
Na, mmol/L	137	130	135	137	136	138	141	139	138
K, mmol/L	4.6	6.2	4.5	3.4	3.4	3.9	3.4	3.5	3.6
tCO2, mmol/L	20.0	14.4	21.0	25.0	25.0	23.0	30.0	28.0	24.0
AST, IU/L	37	91	76	40	35	52	148	46	33
ALT, IU/L	72	82	69	39	36	51	135	105	66
CRP, mg/L	109.0		451.3		301.7		122.2		44.7
Procalcitonin, ng/mL	65.9		41.9						
PMN in peritoneal fluid, /μL	2731	381	131	44	2				
**Inotropic dosage**									
Norepinephrine, mcg/kg/min	0.60	0.80	0.56	0.20	0.12	0.02	0.00		
Vasopressin, IU/min		0.01	0.00	0.00	0.00				

*Abbreviations: HP* hemoperfusion, *WBC* white blood cell, *BUN* blood urea nitrogen, *Cr* creatinine, *AST* aspartate transaminase, *ALT* alanine transaminase, *CRP* C-reactive protein, *PMN* polymorphonuclear neutrophils, *IU* international unit

## Discussion and conclusion

*Vibrio vulnificus* is an opportunistic pathogen belonging to the marine environment. It is usually transmitted by ingestion of infected seafood, especially filter-feeding aquatic organisms such as oysters, mussels, and clams. The incidence of *Vibrio vulnificus* infection has been recently increasing [3]. Wound infections in high-risk individuals may spread rapidly, developing into severe myositis and necrotizing fasciitis. Such serious wound infections require aggressive surgical treatment in addition to proper antibiotics. The mortality rate of primary septicemia is reported to exceed 40%.

Several predisposing medical conditions are known to be associated with a high susceptibility to *Vibrio vulnificus* infection [4]. Renal diseases, including ESRD, are also considered as potential risk factors for *Vibrio vulnificus* infection. Dysfunctions of neutrophils, monocytes, and lymphocytes observed in uremia could play a role in increasing the risk of certain life-threatening infections in these patients [5]. Moreover, *Vibrio vulnificus* is known to grow rapidly in iron rich environments, a situation commonly encountered among dialysis patients who frequently need blood transfusions or iron supplementation to correct anemia [6, 7].

The combination of tetracycline and a third-generation cephalosporin is recommended as the initial antibiotics of choice for *Vibrio vulnificus* infection [8]. In a previous retrospective study, fluoroquinolones were also suggested for treating necrotizing fasciitis caused by *Vibrio vulnificus* [9]. Accordingly, we thought doxycycline plus ceftriaxone with intraperitoneal ciprofloxacin could be an effective combination to treat the septicemia and also prevent the skin lesions of the lower extremities from progressing to fatal necrotizing fasciitis, which would inevitably require emergent operation.

There have been several attempts to verify the efficacy of hemoperfusion using polymyxin B immobilized fiber in patients with septic conditions. Even though the preliminary results of the EUPHAS (Early Use of Polymyxin B Hemoperfusion in Abdominal Sepsis) trial demonstrated that polymyxin B hemoperfusion added to conventional therapy significantly improved hemodynamics and organ dysfunction and reduced 28-day mortality in patients with severe sepsis or septic shock who underwent emergency surgery for intra-abdominal infections [10], the EUPHRATES (Evaluating the Use of Polymyxin B Hemoperfusion in a Randomized controlled trial of Adults Treated for Endotoxemia and Septic shock) study failed to show a beneficial impact of polymyxin B hemoperfusion treatment on patient survival at 28 days among patients with septic shock and high endotoxin activity [11]. Payen et al. also found that there were no significant improvements in mortality and organ failure in peritonitis-induced septic shock patients treated with polymyxin B hemoperfusion [12]. Taken together, there is insufficient evidence to introduce the routine use of polymyxin B hemoperfusion for the treatment of sepsis or septic shock. Nevertheless, it has been suggested that treatment with polymyxin B hemoperfusion stabilizes hemodynamics, which results in less vasopressor requirement and organ dysfunction in patients with abdominal septic shock [10]. On the other hand, it is known that the pathogenicity of *Vibrio vulnificus* arises from its capsular polysaccharide, cytolysin, and lipopolysaccharides [13]. Since the suggested potential action mechanisms of polymyxin B hemoperfusion are neutralization of endotoxin and removal of circulating lipopolysaccharides (key components in sepsis, especially sepsis caused by gram-negative bacteria [14]), hemoperfusion treatment in our case may have prevented the patient from progressing to a worse clinical course by such mechanisms as well as by facilitating hemodynamic stabilization.

In the current case, CVVHDF (continuous venovenous hemodiafiltration) was selected for renal replacement therapy in addition to CAPD due to the rapid exacerbation of hyperkalemia and metabolic acidosis. Continuous equilibrated peritoneal dialysis (CEPD) or high-volume peritoneal dialysis (HVPD) could also be considerable options in sepsis patients who are in hypercatabolic states. However, in the current case, abdominal distension and ileus was aggravating which prevented the application of high volume peritoneal dialysis treatments. The decision to commence CRRT and obtain vascular access also enabled the rapid administration of hemoperfusion.

To date, there have been two reported cases of CAPD peritonitis caused by *Vibrio vulnificus* [15, 16] and 1 case of *Vibrio vulnificus* peritonitis followed by severe septicemia and necrotizing fasciitis in a patient undergoing CAPD [17], all of which recovered with adequate antibiotics and surgical treatment, if needed. Furthermore, there have been 2 non-ESRD cases of septic shock caused by *Vibrio vulnificus* which were successfully treated by polymyxin B hemoperfusion [13, 18]. The unique finding of our patient was that CAPD peritonitis caused by *Vibrio vulnificus* followed by septic shock was effectively treated with proper antibiotics plus the use of polymyxin B hemoperfusion. With the deterioration of the septic shock, leukopenia was observed temporarily. This transient finding was thought to be caused by the fulminant infection. Several previous reports have also shown *Vibrio vulnificus* infection to be accompanied with leukopenia. The presence of leukopenia in these patients was found to be associated with poor prognosis [19, 20]. In addition, although the skin lesions evolved to erythematous bullae, they did not aggravate to necrotizing fasciitis, so invasive surgical intervention, such as fasciotomy or debridement, was not necessary.

There are several limitations of this case report. First, the main reason for the initial hemodynamic stabilization cannot be completely specified. Hemoperfusion had been initiated with concurrent change of antibiotics in the present case. Since stabilization of hemodynamics had been observed after a relatively short time after antibiotics regimen change and hemoperfusion administration, it could be assumed that hemoperfusion may have played a bigger role compared to the changed antibiotics. Previous reports do report cases of *Vibrio vulnificus* sepsis in PD patients treated with a combination of ciprofloxacin and third generation cephalosporin without hemoperfusion. However, although these reports do not specify the time interval between antibiotics treatment and blood pressure stabilization, they do describe the period to clinical improvement to be in the range from few days to weeks compared to the several hours of the current case. Secondly, the actual endotoxin levels before and after hemoperfusion treatment were not available. Unfortunately, assays measuring the amount of endotoxin such as EAA (Endotoxin Activity Assay) are not yet available in Korea and hemoperfusion is prescribed empirically in hemodynamically unstable gram-negative sepsis patients.

In conclusion, thorough history taking and suspicion of *Vibrio vulnificus* infection in susceptible immunocompromised patients are essential for early diagnosis and proper management, which can dramatically improve clinical outcomes. It would be worthwhile to acknowledge that peritonitis can be an early clinical sign of *Vibrio vulnificus* infection in CAPD patients, and polymyxin B hemoperfusion should be recognized as a treatment option in such patients.

## Data Availability

All available information is contained within the present manuscript.

## Abbreviations

CAPD: Continuous ambulatory peritoneal dialysis
CRRT: Continuous renal replacement therapy
CKD: Chronic kidney disease
AKI: Acute kidney injury
WBC: White blood cell
PMN: Polymorphonuclear
CRP: C-reactive protein
PD: Peritoneal dialysis
ICU: Intensive care unit
ESRD: End-stage renal disease
SOFA: Sequential Organ Failure Assessment
APACHE-II: Acute Physiology and Chronic Health Evaluation-II
SAPS-II: Simplified Acute Physiology Score-II
CVVHDF: Continuous venovenous hemodiafiltration
CEPD: Continuous equilibrated peritoneal dialysis
HVPD: High-volume peritoneal dialysis
EAA: Endotoxin Activity Assay
IP: Intraperitoneal
IV: Intravenous
PO: Per os
